# Non-opaque soft tissue foreign body: sonographic findings

**DOI:** 10.1186/1471-2342-11-9

**Published:** 2011-04-10

**Authors:** Afshin Mohammadi, Mohammad Ghasemi-Rad, Maryam Khodabakhsh

**Affiliations:** 1 Radiology Department, Urmia University of Medical Sciences, Urmia, Iran; 2 Student research committee, School of Medicine, Urmia University of Medical Sciences, Urmia, Iran; 3 School of Medicine, Urmia University of Medical Sciences, Urmia, Iran

## Abstract

**Background:**

Soft tissue foreign bodies are a common cause of orthopedic consultation in emergency departments. It is difficult to confirm their existence because conventional radiology only detects radio-opaque foreign bodies. Sonography can be a useful diagnostic method. The aim of this study is to evaluate diagnostic accuracy of sonography in detection and localization of non-opaque foreign bodies.

**Methods:**

We evaluated 47 patients with suspected foreign body retention in soft tissues by 10 MHz linear array transducer. A single radiologist performed all examinations with 6 years' experience in musculoskeletal Sonography. We detected and localized the presence of the foreign body in the soft tissue as guidance for facilitating the surgery.

**Results:**

We detected soft tissue foreign body in 45 cases as hyperechoic foci. Posterior acoustic shadowing was seen in 36 cases and halo sign was seen in 5 cases due to abscess or granulation tissue formation. Surgery was performed in 39 patients and 44 foreign bodies were removed.

**Conclusion:**

Sonography is a useful modality in detection and localization of radiolucent foreign bodies in soft tissue which can avoid misdiagnosis during primary emergency evaluation.

## Background

Penetrating object injuries are a common problem in the emergency department, and retained foreign bodies in soft tissues complicate many such injuries. Because a retained foreign body may cause severe infection or inflammatory reaction, detection and removal of foreign bodies are necessary [1].

Punctured wounds and soft tissue lacerations are inspected, palpated and explored to rule out the presence of a foreign body, and radiographic evaluations are routinely obtained to confirm radio-opaque foreign bodies such as glass, metal, and stone within the soft tissue [2,3], However 38% of such foreign bodies are overlooked at initial examination in the emergency room [4].

A radiolucent foreign body such as wood frequently remains undetected [3]. In such situations, other imaging modalities are needed for diagnosis. Sonography plays an important role in the evaluation of these patients [5].

Sonography has a reported sensitivity of 95% for detection of foreign bodies [6,7].

In previous reports the positive predictive value of Conventional Radiography (CR) and Sonography (US) were 100% and 95% respectively and for Computed Tomography (CT) and Magnetic Resonance Imaging (MRI) were 95% and 93.8% respectively. CT had a negative predictive value of 78.3%, while US, MRI, and CR had 73.7%, 70.1%, and 53.7%, respectively [8].

Non-opaque foreign bodies are visualized as hyper-echoic foci with accompanying acoustic shadows [5]. This shadow may be either complete or partial depending on the angle of insonation and the composition of the foreign body [4]. A hypoechoic halo surrounding the foreign body is sometimes seen, which represents edema, abscess or granulation tissue [9].

The purpose of the study was to determine effectiveness of Sonography for detection of radiolucent foreign bodies and to summarize all clinical experiences using Sonography in the management of patients with a suspected retained foreign body.

## Methods

Forty-seven patients were referred for Sonographic examination because of possible retention of soft tissue foreign bodies in the upper or lower extremities during a 3-year period (January 2006 to January 2009). General physicians of the emergency department of Imam Khomeini University Hospital, Urmia, Iran, referred 44 male patients and 3 female patients. All patients had undergone plain X-rays that were negative for foreign bodies. Mean age was 26 years (Range 12 to 44). Those patients having X-rays negative for foreign bodies were referred for Sonographic evaluation.

We evaluated the site of penetrating trauma and location of the patients' chief complaint both longitudinally and transversely by high frequency Sonographic scanning.

A radiologist with 6 years experience in musculoskeletal system obtained all Sonography (Esaote MyLab 50, Genova, Italy).

For obtaining a good soft tissue resolution, we need a high frequency linear transducer which has been shown to be helpful in detecting small foreign bodies [10].

In all patients, the contra-lateral extremity was examined as a comparison. Whenever a foreign body was localized, its length and depth beneath the skin was measured with computerized calipers.

We evaluated the Sonographic findings of various soft tissue foreign bodies, such as posterior acoustic shadowing, posterior comet tail, and a halo sign in all patients.

The University research and ethics committee approved the study protocol; written informed consent was obtained from all patients.

## Results

We detected and localized the foreign bodies in 45 of the 47 patients by Sonography. The sensitivity and specificity of Sonography in comparison with surgery in diagnosis of soft tissue foreign bodies was 100%.

Surgery revealed that 20 objects were wooden particles and date rose thorns; 6 objects were fish bones in fishmonger and 18 objects were broken glass particles (39 patients with 44 objects).

Thirty-nine foreign bodies that were diagnosed by Sonography under local anesthesia were surgically removed. All patients were symptom free during follow-up with no further complications recorded during observation.

Six patients had small foreign bodies with minor symptoms and without impairment of limb function. All 6 patients opted for regular follow-ups instead of surgical removal. The patients were symptom free after 3 months of follow-up. Two patients had negative Sonographic findings that were followed-up with non-surgical conservative treatment.

The smallest foreign body was a glass object detected in the forearm which measured 3 mm in length. The mean ± SD length of detected foreign body was 7.9 ± 0.6 mm. Wooden objects were the most common type.

We detected foreign bodies in toes (6 patients), forearms (7 patients), fingers (13 patients), soles of the foot (16 patients) and calves (3 patients) by Sonography.

One of the patients had 4 pieces of foreign body in his foot and one patient had 3 pieces of foreign body in his finger.

Surgery was performed in 39 patients and 44 foreign bodies were removed.

**Figure 1:**
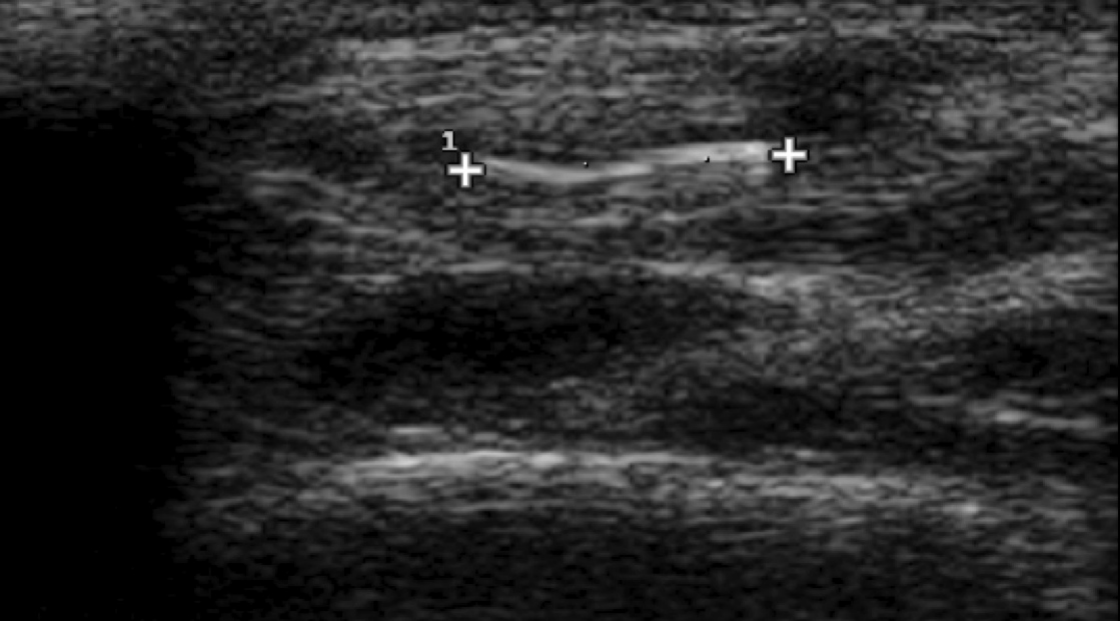
**Longitudinal sonogram shows a hyperechoic 4 mm long broken glass (long arrow) without posterior acoustic shadowing in the forearm of a 24 years old man**.

**Figure 2:**
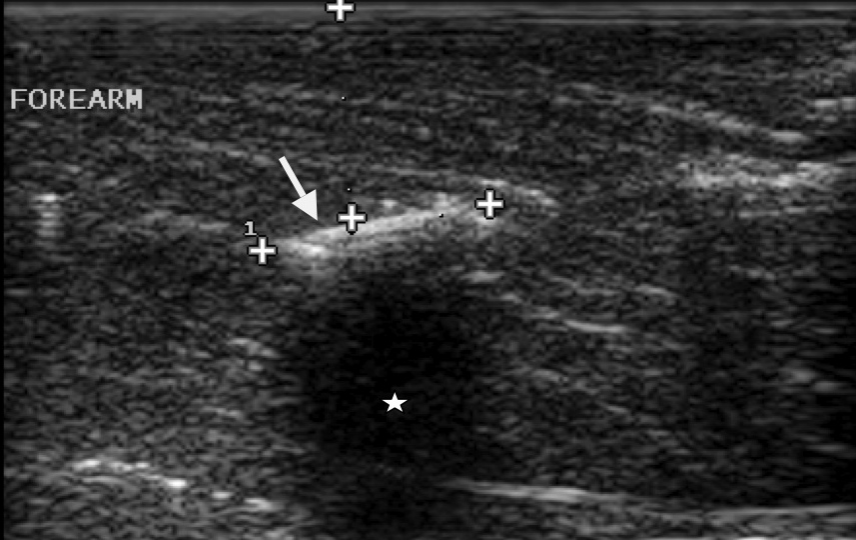
**Longitudinal sonogram shows a hyperechoic 2 cm long wooden foreign body (long arrow) with posterior acoustic shadowing (satellites) forearm of 21 years old man**.

**Figure 3:**
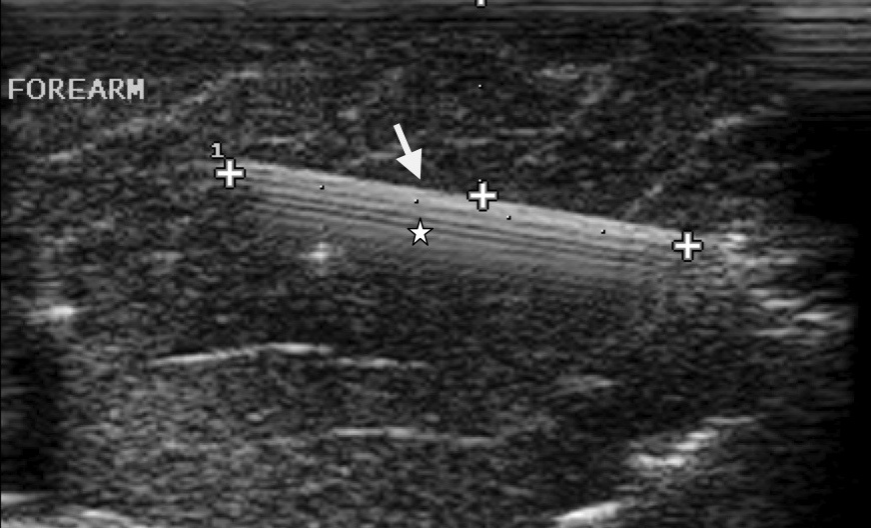
**Longitudinal sonogram shows a hyperechoic 5 cm long broken glass foreign body (long arrow) with posterior comet tail (satellites) in forearm of 21 years old man**.

Sonography revealed the foreign body as hyper-echoic objects with or without posterior acoustic shadowing in all 45 patients. (Figures 1, 2&3)

**Figure 4:**
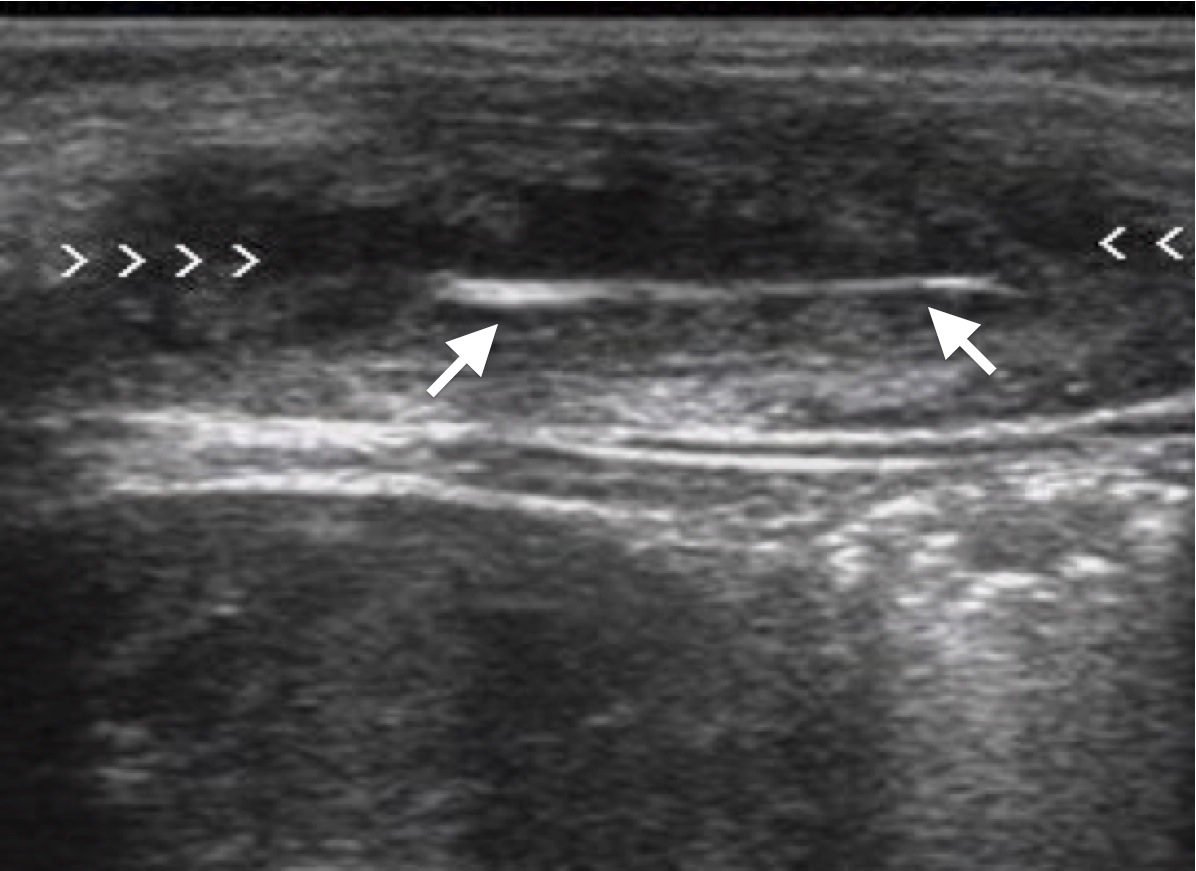
**Longitudinal sonogram shows a hyperechoic 6 mm wooden foreign body (long arrow) without posterior acoustic but with surrounding halo sign (arrowhead) due to soft tissue abscess in the forearm**.

Sonography revealed the foreign body as the late complication of previous penetrating trauma with sustained pain and tenderness on trauma site in 5 patients with hypo-echoic mass surrounding the foreign objects due to abscess and granulation tissue formation. (Figure 4).

We detected posterior acoustic shadowing in 15 cases of wooden objects; Rose thorns in 21 patients with wooden foreign bodies. Posterior acoustic shadowing or comet tail sign was not seen in 6 patients. The mean ± SD length of detected wooden foreign bodies with posterior acoustic shadowing was 9.8 ± 0.4 mm and 4.6 ± 0.3 mm without posterior acoustic shadowing.

We detected posterior acoustic shadowing in 15 out of 18 patients with broken glass objects and posterior comet tail sign in 2 patients (50 mm and 9 mm foreign bodies in length respectively). Posterior acoustic shadowing or posterior comet tail sign was not detected in one patient (4 mm foreign body in length).

We detected posterior acoustic shadowing in all fish bone foreign bodies (6 patients). The mean ± SD length of detected fish bones with posterior acoustic shadowing was 6.9 ± 0.6 mm.

The type of foreign body, its mean ± SD measure, and the body locations are expressed in table 1.

**Table 1:** Summerized the type of foreign body, its mean measurements and the location

	***Type of FB***		
***Site of FB***	***Wooden FB*** ***MD: 7.2 mm***	***Glass*** ***MD: 9.4 mm***	***Fish bone*** ***MD: 6.9 mm***
***Sole of Foot***	***10***	***5***	***-***
***Finger***	***1***	***8***	***4***
***Forearm***	***4***	***1***	***2***
***Toe***	***3***	***3***	***-***
***Calves***	***2***	***1***	***-***
***All***	***20***	***18***	***6***

FB: Foreign Body, MD:Mean Diameter.

## Discussion

A retained foreign body in the soft tissues of extremities is not very common. Diagnosis requires high index of suspicion. Exclusion of its presence is important, given the possible allergic, inflammatory, and infectious complications associated with a retained foreign body [1].

Conventional radiographs should be obtained to rule out the presence of radio-opaque foreign objects. Plain radiographs will depict approximately 80% of all foreign bodies, but several types of radiolucent foreign bodies such as wood remain undetected [11]. Plain radiographs of wooden FB are negative in 86% of such patients [4]. In these patients Sonography is the modality of choice for identification of such radiolucent FB.

The identification of wooden foreign bodies may be difficult on MRI, especially when foreign bodies are small and there is no associated abscess, granulation tissue, or fluid collection. In such cases, the foreign body may appear as a signal void with surrounding nonspecific granulation tissue. Wooden foreign bodies may be seen signal void in all sequences, but after water absorption it could be seen hypo-intense on T1 and hyper-intense on T2 images [5].

When compared with Sonography, MRI is more expensive, less readily available, and has less value in the detection of small wooden foreign bodies. Likewise, MRI has obvious limitations for the evaluation of patients in the emergency room.

Sonographic evaluation provides important information on the depth, size and anatomical relationship with surrounding structure [6,9], and [12]. Although CT has sensitivity 5-15 times greater than that of plain X -ray, it is not as sensitive as US, or MRI [2]. Additionally, the expense, use of radiation, and availability make the use of CT less than optimal in the clinical setting.

Surgical dissection is facilitated by accurate knowledge of location of the FB related to muscles, tendons and vessels. Detection of foreign body is difficult in interphalangeal space and in air contaminated tissue after a penetrating trauma. FB must be distinguished from hyper-echoic body tissue such as ossified cartilage sesamoid bones, scar tissue, gas bubble, intermuscular fascia etc. Acoustic shadowing is an important clue in the differential diagnosis [6,9]. Acoustic shadowing can differentiate foreign body from scar tissue, gas bubble and normal intermuscular fascia, because they are void of acoustic shadowing.

Our results demonstrate the effectiveness of Sonography for detection of radiolucent FB. It is therefore an important modality that facilitates removal of the object by enabling a shorter exploration with less iatrogenic tissue damage.

Peterson JJ et al [5] showed that Sonography is the modality of choice in patients who present with a history of antecedent skin puncture or when a penetrating injury is suspected.

We detected the posterior acoustic shadowing in 15 out of 20 wooden objects that was similar to the previous study [13] that demonstrated posterior acoustic shadowing in only 11 out of 17 cases of wooden FB. This is perhaps because of the orientation of the FB relative to the sound bean and chronicity of the retained FB. Retained wooden FB absorbs fluid, which alters its imaging characteristics [1].

Fornage BD et al [14] showed that retained wooden foreign bodies are easily identified, with the leading edge of the echogenic wood resulting in marked acoustic shadowing.

Jacobson JA [15] showed that Sonography could be used effectively to locate wooden foreign bodies as small as 2.5 mm in length.

Dumarey A et al [16] showed that CT gave a good anatomic overview, but was not able to show the smaller fragments. Performing Sonography is mandatory in patients with penetrating injuries by foreign bodies because it is very sensitive.

We detected the posterior comet tail sign in only 2 out of 18 patients with broken glass objects in soft tissue.

The depth of all foreign bodies was smaller than 4 cm, because all of them were embedded in the distal part of upper and lower limbs.

We believe that all foreign bodies were seen during Sonographic examination as echogenic objects and most of them (wooden, glass, and etc...) may also show similar Sonographic findings. Most of them show posterior acoustic shadowing.

## Conclusion

In conclusion, Sonography can be used effectively to locate radiolucent FB with high certainty, and should be considered for patients suspected of having a FB in the setting of negative X-rays. US can be used as a modality of choice in the emergency department to avoid missed, or under diagnosis of retained foreign bodies.

## Competing interests

The authors declare that they have no competing interests.

## Authors' contributions

AM participated in the idea, planning, data analysis and interpretation, statistical analysis, and writing the report

MG participated in data collection; follow up, analysis and drafting

MK participated in the data analysis and interpretation, statistical analysis. All authors read and approved the final manuscript.

## Pre-publication history

The pre-publication history for this paper can be accessed here:

http://www.biomedcentral.com/1471-2342/11/9/prepub

## References

[B1] Mohamadi A Kodabakhsh M Wooden foreign body in lung parenchyma, a case report Turkish Journal of trauma and emergency surgery 2010 16 5 480 482 21038131

[B2] Flom LL Ellis GL Radiologic evaluation of foreign bodies Emerg Med Clin North Am 1992 10 163 176 1732094

[B3] Graham DD Jr Ultrasound in the emergency department: detection of wooden foreign bodies in the soft tissues J Emerg Med 2002 22 1 75 9 10.1016/S0736-4679(01)00440-1 11809560

[B4] Anderson MA Newmeyer WL Kilgore ES Diagnosis and treatment of retained foreign bodies in the hand Am J Surg 1982 144 63 67 10.1016/0002-9610(82)90603-1 7091533

[B5] Peterson JJ Bancroft LW Kransdorf MJ Wooden foreign bodies: imaging appearance AJR Am J Roentgenol 2002 178 3 557 62 11856673 10.2214/ajr.178.3.1780557

[B6] Crowford R Matheson AB Clinical value of ultrasonography in detection and removal of radiolucent foreign bodies Injury 1989 20 341 343 10.1016/0020-1383(89)90008-9 2697696

[B7] Ober CP Jones JC Larson MM Lanz OI Werre SR Comparison of ultrasound, computed tomography, and magnetic resonance imaging in detection of acute wooden foreign bodies in the canine manus Vet Radiol Ultrasound 2008 49 5 411 8 10.1111/j.1740-8261.2008.00399.x 18833946

[B8] Venter NG Jamel N Marques RG Djahjah F Mendonça Lde S Evaluation of radiological methods for detection of wood foreign body in animal model Acta Cir Bras 2005 20 Suppl 1 34 41 16186971

[B9] Little CM Parker MG Callowich MC Sartori JC The ultrasonic detection soft tissue foreign bodies Invest Radiol 1986 21 275 7 10.1097/00004424-198603000-00014 3514541

[B10] Walter JP Physics of high resolution -practical aspect Radiol Clin North Am 1985 23 3 11 3883405

[B11] Donaldson J Radiographic imaging of foreign bodies in the hand Hand Clin 1991 7 125 134 2037630

[B12] Conti RJ Shinder M Soft tissue calcification induced by local corticosteroid injection J Foot Surg 1991 30 34 7 2002185

[B13] Gilbert FJ Campbel RSD Bayliss AP The role of ultrasound in detection of non- opaque foreign bodies Clin Radiol 1990 40 109 112 10.1016/S0009-9260(05)80140-0 2407413

[B14] Fornage BD Schernberg FL Sonographic diagnosis of foreign bodies of the distal extremities AJR 1986 147 567 569 3526845 10.2214/ajr.147.3.567

[B15] Jacobson JA Powell A Craig JG Bouffard JA van Holsbeeck MT Wooden foreign bodies in soft tissue: detection at US Radiology 1998 206 1 45 8 9423650 10.1148/radiology.206.1.9423650

[B16] Dumarey A De Maeseneer M Ernst C Large wooden foreign body in the hand: recognition of occult fragments with ultrasound Emerg Radiol 2004 10 6 337 9 15278719 10.1007/s10140-004-0333-8

